# Improving COVID‐19 Vaccine Uptake Among Ethnic Minority Communities in Wales: A Community‐Based Approach

**DOI:** 10.1111/imcb.70140

**Published:** 2026-06-30

**Authors:** Bnar Talabani, Sayma Hanif, Kasim Ramzan, Farzana Mohammed, Amara Naseem, Emaad Alauddin

**Affiliations:** ^1^ Renal Medicine, Bristol Renal, Translational Health Sciences University of Bristol Bristol UK; ^2^ General Practitioner Cardiff UK; ^3^ Health Education & Improvement Wales Cardiff UK

## Abstract

During the COVID‐19 pandemic, misinformation and vaccine hesitancy disproportionately affected ethnic minority communities across the United Kingdom, including in Wales. Muslim Doctors Cymru (MDC), a grassroots coalition of Muslim healthcare professionals, played a pivotal role in countering this challenge. This paper describes the strategies and impact of MDC's work in addressing health misinformation, building trust, and promoting vaccine uptake among ethnic minority communities in Wales. Through culturally sensitive engagement, multilingual public health messaging, and close collaboration with mosques, community leaders, and local media, MDC delivered targeted outreach that bridged gaps between public health authorities and underserved populations. This case study draws on a grassroots‐efforts approach to tackling vaccine inequity using community‐based approaches. Findings highlight the importance of culturally competent healthcare communication and the value of community‐led initiatives in improving public health outcomes during crises. MDC's model offers a replicable framework for addressing health inequalities and misinformation in diverse populations.

## Graphical abstract

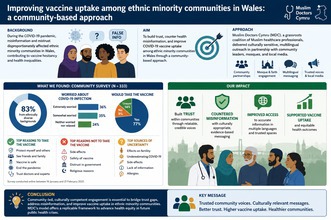

## 1 Introduction

Misinformation during the COVID‐19 pandemic had a significant negative impact on people's behavior and attitudes towards public health restrictions and vaccination rates. False and misleading claims spread rapidly—especially via social media—and led to widespread confusion, fear, and mistrust [1]. Often shared by non‐health workers and non‐scientists, this created the perfect storm for non‐compliance with evidence‐based measures, rendering specific communities vulnerable to the virus. This was compounded by prominent figures from within the scientific and medical community, spreading disinformation and misinformation to dissuade vaccine uptake. This led to vaccine hesitancy, with many either refusing or delaying their COVID‐19 vaccine uptake, while others did not have the full‐recommended vaccination courses. Myths around fertility, speed of vaccine development and harmful substances contributed to this [1, 2, 3]. As a result of this dis‐ and misinformation, a perpetuated cycle of mistrust in government and healthcare organizations was exacerbated, undermining public health policy. Conspiracy theories snowballed predominantly on social media, resulted in concern and hesitancy among many, including those who usually follow medical and scientific advice [1, 2, 3]. This culminated in a hampering of efforts to control the pandemic, prolonging the spread of the virus, leading to more variants, undermining efforts to achieve herd immunity [4].

**FIGURE 1:**
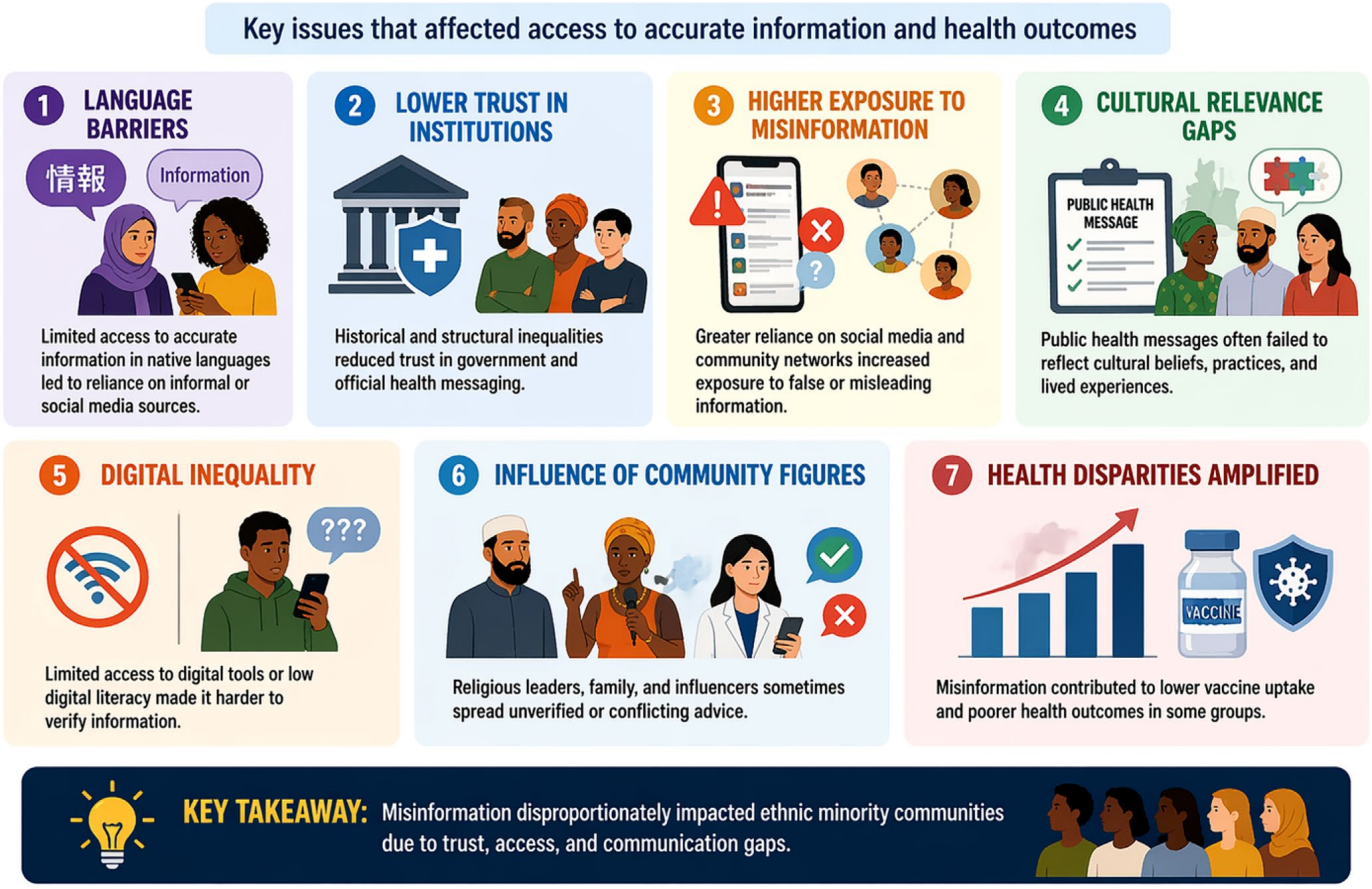
Schematic diagram summarizing different factors contributing to vulnerability of health misinformation in people from ethnic minority backgrounds.

Ethnic minority communities were particularly at risk. During the COVID‐19 pandemic, ethnic minority groups in the UK experienced disproportionately higher rates of infection, hospitalization, and mortality compared to the White British population [5, 6]. This disparity was influenced by a combination of socioeconomic factors, occupational exposures, pre‐existing health conditions, barriers to healthcare access, lack of trust in authority, and dis‐ and misinformation [4] (Figure 1). Over the entire pandemic period, COVID‐19 mortality rates for males were highest among the Bangladeshi and Pakistani groups [5]. For females, rates were also highest for these groups, though not significantly higher than the Gypsy and Irish Traveler group or other Black groups [5].

Dis‐ and misinformation were particularly targeted at these communities, with tangible impact. They were spread predominantly on social media platforms [7], were available in several languages, and perpetuated by seemingly trusted sources, such as health workers, scientists, faith leaders, and other community figures who were influential among their communities [7]. There was a stark void of accurate information in these languages and in these spaces to combat this. This was further compounded by language barriers and a lack of health literacy among some people in minority ethnic communities: health messaging to combat misinformation was initially only available in English, and when this was later rectified, written resources were created in languages other than English, which failed to take into account issues with health literacy in some communities and among individuals. Furthermore, a distrust in government, authorities, and media (perpetuated by negative portrayal [8] of people from Black, Asian and other minority ethnic communities as well as longstanding inequalities) devalued any official communication during this time. The absence of relatable, trusted voices from within these communities in mainstream health communication further widened the gap between public health authorities and ethnic minority groups (Figure 1).

Witnessing the impact of dis‐ and misinformation go unchallenged in ethnic minority communities in Wales, we formed Muslim Doctors Cymru (MDC), comprising a volunteer group of healthcare professionals in Wales. We played a pivotal role in combating vaccine misinformation, particularly within ethnic minority communities. Founded in January 2021 by Cardiff‐based doctors, MDC aimed to address the high levels of concern and hesitancy towards COVID‐19 vaccines among these communities, fueled by misinformation in various languages and a lack of accessible, accurate information. Our model was simple; we used trusted voices and trusted spaces to deliver accurate, evidence‐based health messaging. Our goal was to facilitate informed decision‐making rather than tell people what they should do.

## 2 COVID‐19 Infection & Vaccine Survey

**FIGURE 2:**
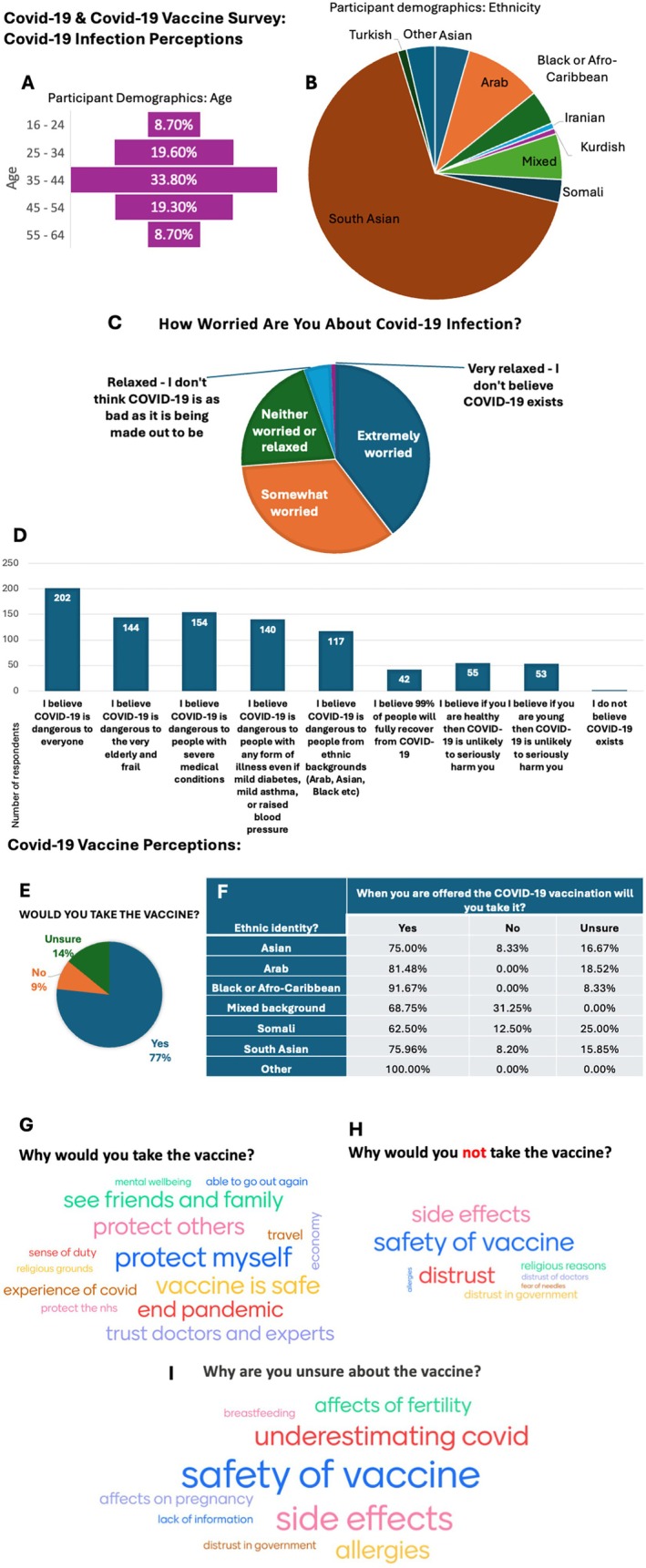
Summary of COVID‐19 infection and vaccine survey results. (A) Participant demographics according to age. (B) Participant demographics according to ethnicity. (C) Summary of response to “How worried are you about COVID‐19 infection.” (D) Summary of attitudes about COVID‐19 infection. (E) Summary of response to who would take COVID‐19 vaccination. (F) Breakdown of responses regarding decision to vaccinate according to ethnicity. (G) Word‐cloud summary of responses to “Why would you take the vaccine?” (H) Word‐cloud summary of responses to “Why would you not take the vaccine?” (I) Word‐cloud response to “Why are you unsure about the vaccine?”

We sought to understand attitudes towards vaccination among individuals from our target population. We achieved this by conducting an anonymous online survey. This was disseminated predominantly via our social media platforms and among contacts on WhatsApp. The survey was open to participants between the 16th of January (approximately two weeks after the first SARS‐CoV‐2 vaccines were approved in the UK) and the 21st of February 2021 (Figure 2). A total of 333 respondents participated, providing a diverse range of perspectives shaped by cultural, religious, and personal experiences; 275 were from ethnically diverse backgrounds and 58 respondents were Caucasian. 47% of participants were female. The majority of participants were aged 34–44 years old. The majority were from South Asian descent (which included people from Pakistani, Indian, and Bangladeshi origins). Regarding attitudes about the severity of COVID‐19 infection, 6% of participants were either relaxed/very relaxed about COVID‐19 infection risk and did not think it was a risk to them, and this was more prevalent among younger participants, but not limited to these age groups. Approximately 23% were neither worried nor relaxed about infection risk and participants displayed a range of attitudes about COVID‐19 infection risk. When asked about COVID‐19 vaccines, 77% reported they would get vaccinated, while 14% remained unsure and 9% had decided against vaccination. Of those unsure/adamant against vaccination, the majority were from Somali and Mixed backgrounds. When participants were asked why they wanted to receive their COVID‐19 vaccination, the majority replied that they wanted to protect themselves and others as well as work towards ending the pandemic. Some indicated trust in doctors and experts. Of those that had decided against vaccination, the biggest concern was the safety of the vaccine, followed by distrust and concerns around vaccine side effects. Those who had expressed vaccine hesitancy reported their concerns around safety and vaccine side effects; however, a significant number of respondents had underestimated the impact of COVID‐19 infection. The responses of this survey formed the basis of our campaign to address vaccine hesitancy.

Overall, the findings suggested a generally positive view of vaccines, with many recognizing their importance in protecting individual and public health. However, the survey also highlighted areas of concern, including hesitancy driven by dis‐ and misinformation, distrust in healthcare systems, and uncertainty about vaccine safety and side effects (Figure 2). Respondents expressed a need for clearer, culturally sensitive communication and engagement from trusted community and healthcare leaders, which was echoed in comments on our social media posts. The results underpinned the importance of targeted outreach and education to address misconceptions and improve vaccine confidence within these communities.

**FIGURE 3:**
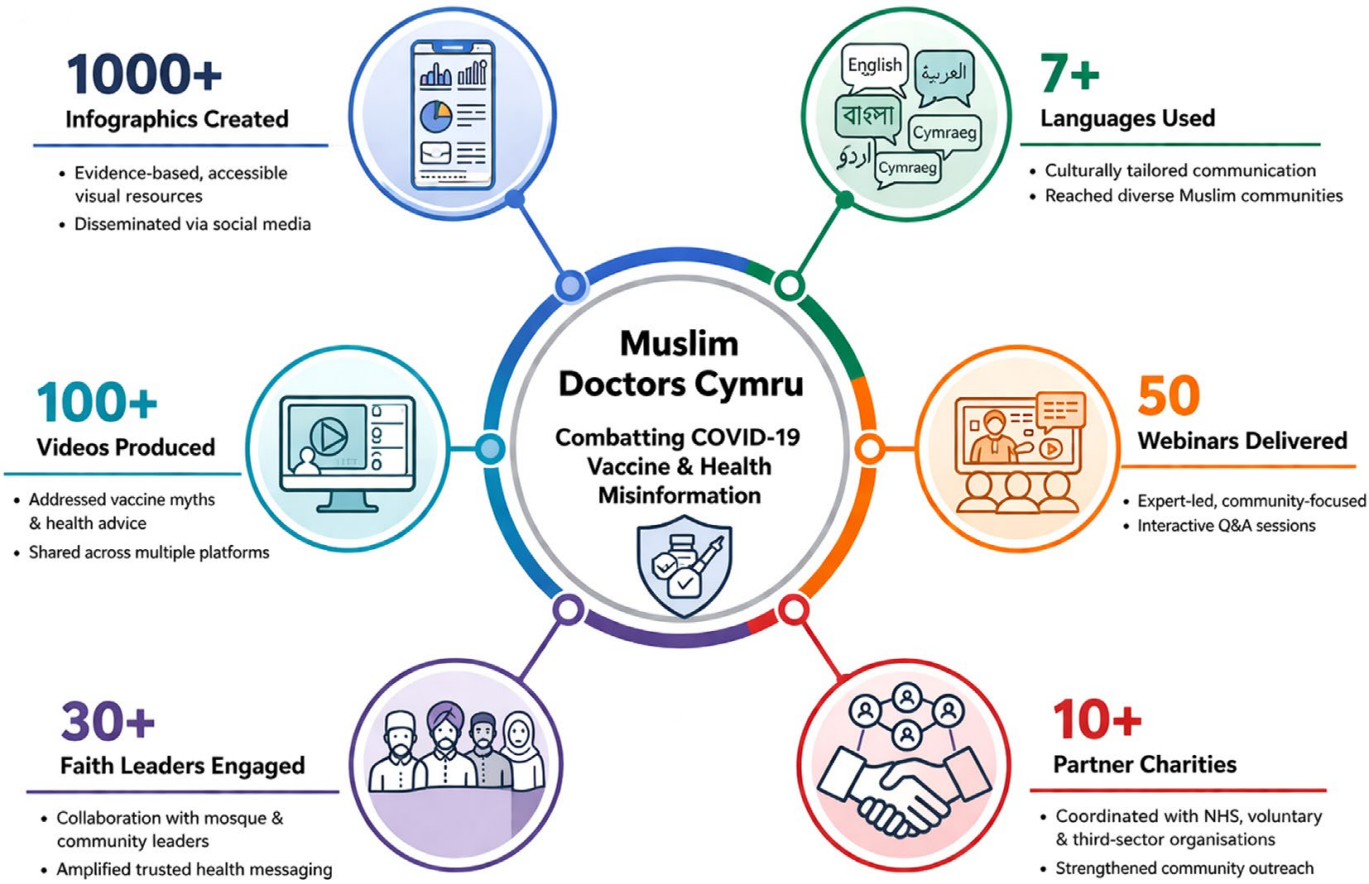
A summary of the social media campaign led by Muslim Doctors Cymru.

Figure 3 schematically summarizes our outreach work on social media. Recognizing the language barriers that hindered effective communication and engagement with BAME communities, we produced and disseminated accurate information in multiple languages, including English, Urdu, Arabic, Bangali Somali, and Kurdish. We utilized social media platforms to share myth‐busting videos and public health messages, ensuring that communities received information in their native languages from trusted voices—comprising health care workers who live locally to our target population. These videos and resources were also shared among contacts on WhatsApp and other messaging platforms to ensure a wide reach. We received excellent feedback on these, with many in our communities sharing with families abroad to provide them with access to accurate information. This included individuals who had adamantly decided against vaccination and had actively worked to dissuade and prevent family members from receiving their vaccinations to changing their minds completely. Not only had they decided to receive their vaccinations and encourage family and friends to do the same, but they played an active role in disseminating our social media posts and videos resources to encourage others outside their sphere to get vaccinated.

## 3 COVID‐19 Vaccine Webinars

**FIGURE 4:**
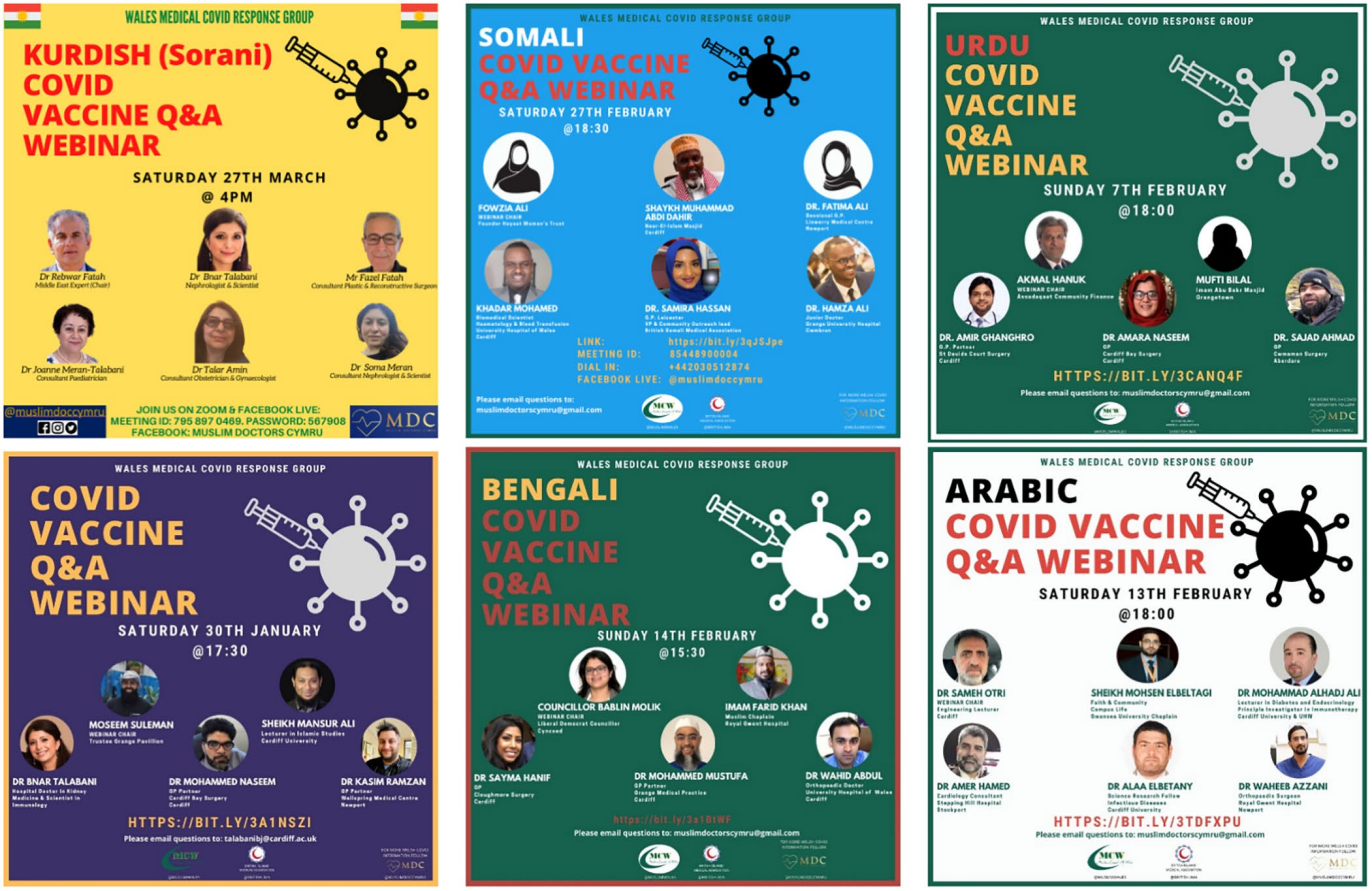
Poster infographics created to advertise COVID‐19 vaccine webinars on social media.

To further engage with communities, MDC organized webinars in various languages, addressing a range of health issues and vaccine‐related concerns. These sessions provided a platform for individuals to ask questions and receive answers in a language they were comfortable with, fostering trust and understanding. Hosted by a well‐respected and known community figure, such as the former head of The Bangladeshi Women's Association. These webinars were tailored to specific minority ethnic communities, with the presence of local faith leaders from these communities to further support health and scientific messaging in a way that was engaging and respectful of cultural nuances. Delivered by MDC volunteers, who had received training and teaching on up‐to‐date scientific evidence on COVID‐19 vaccinations, these webinars were well attended, with attendances ranging from 200–350 people. They were also uploaded onto YouTube and widely shared for later viewing. One of these videos, recorded in Kurdish, was viewed over 958 times in the UK and abroad. Webinars were delivered in English, Urdu, Arabic, Bengali, Kurdish, and Somali (Figure 4). These webinars offered safe spaces whereby individuals could either openly or anonymously ask their questions, which were answered by health professionals and faith leaders, providing further reassurance and support.

## 4 Social Media Videos

In addition to short educational videos on COVID‐19 vaccines, challenging the most common vaccine myths delivered by doctors and scientists, MDC also collaborated with faith leaders, charities, and local businesses to develop community‐based campaigns, inviting community members to create short videos explaining why they had decided to get vaccinated. This created a positive messaging stream around vaccination and empowered individuals to protect themselves, their families, and their communities. We wanted to ensure that our messaging and resources were as far reaching as possible, and helped as many communities as possible, in a way that was respectful of those from all and no faiths, as well as from diverse communities. Therefore, we collaborated with several organizations and charities. These included the Interfaith Council, the UK Black Pharmacists association, Welsh Government, Public Health Wales, and others.

## 5 Mosque Vaccination Pop‐Ups

**FIGURE 5:**
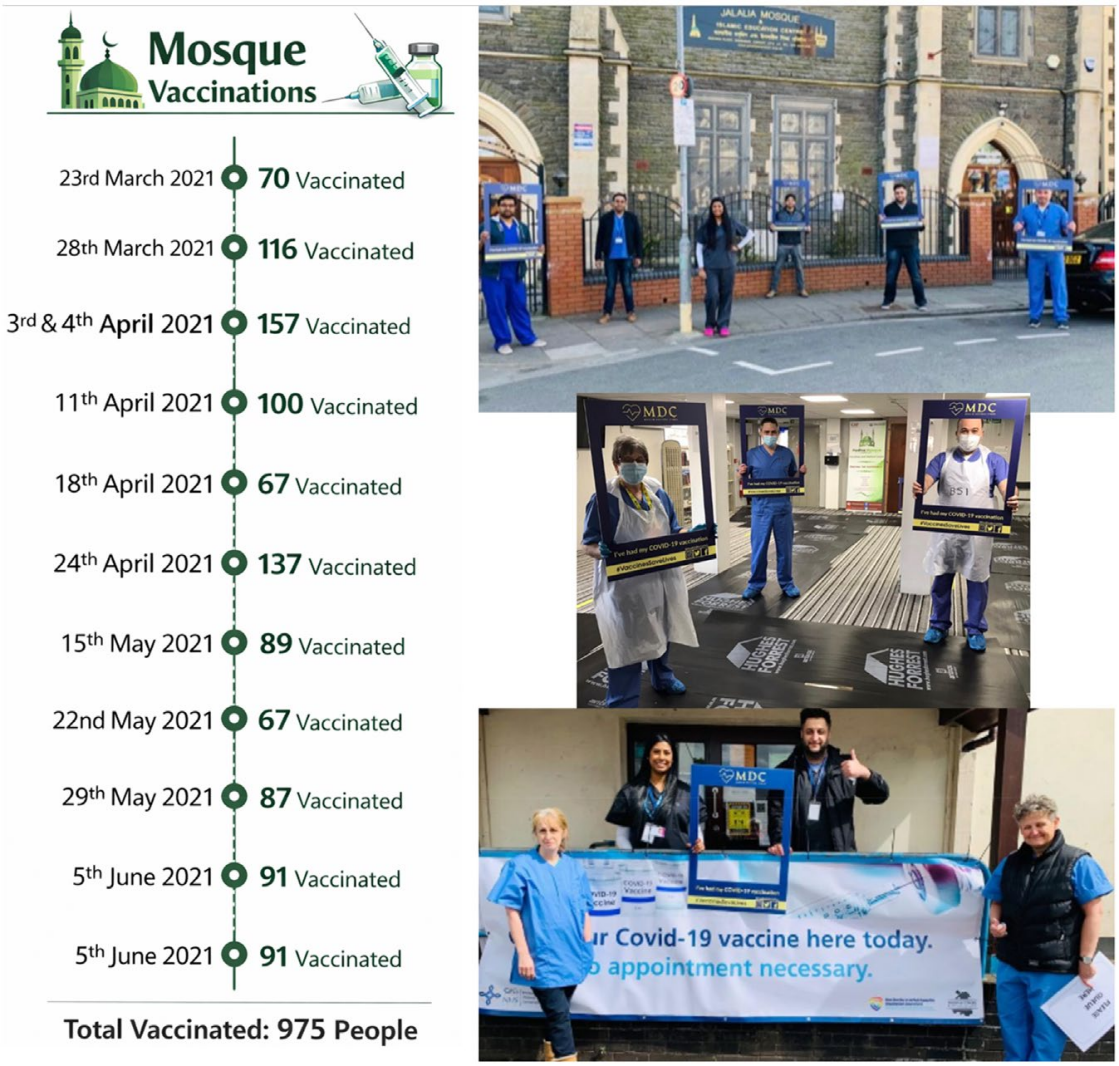
Summary of mosque vaccine pop‐up vaccination rates.

We had adopted a “trusted voices” approach to engaging with BAME communities but wanted to deliver our work in “trusted spaces.” Therefore, we worked with local health boards in Wales to set up the first pop‐up vaccine centers in 6 mosques in South Wales and other trusted settings, making vaccines more accessible and addressing hesitancy by providing vaccinations in familiar and trusted environments. In addition to providing vaccinations in these settings, volunteers from our team were readily available to discuss any concerns with people who had any questions. All were welcome to attend and have discussions, and those who were eligible could get vaccinated. Importantly, this was available to anyone who was eligible to receive their vaccination (either based on age or clinical vulnerability status) but had not yet attended a walk‐in appointment to do so, meaning they had the opportunity to receive their vaccination prior to these pop‐ups but had chosen not to. Vaccinations were delivered by trained staff from local health boards. MDC volunteers (comprising local doctors) were on hand to discuss any concerns where individuals were hesitant, to address their questions. We hosted weekly vaccination pop‐ups between March and June 2021 and vaccinated over 900 individuals (Figure 5). This provided a safe space for individuals to voice and discuss their concerns with qualified individuals. During our first mosque vaccine pop‐up, we were approached by a homelessness charity, whose service users could not access vaccinations in the mass‐vaccination centers as they were not registered with GPs. We vaccinated 58 homeless individuals and communicated this issue back to Welsh Government, who subsequently launched their “Nobody Left Behind Campaign” to ensure anyone could walk into any vaccination center and receive their COVID‐19 vaccination, without facing any barriers.

## 6 Collaborations With Government, Health Organizations & Media Outlets

Furthermore, we engaged with local government to provide insight to vaccine hesitancy among ethnic minority communities in Wales. We shared the results of our survey with Welsh Government and Public Health Wales and were invited to attend the Welsh Government's monthly vaccine communication strategy meetings. We used this platform to share our experiences at the frontline. We also worked closely with media outlets, giving regular interviews on national radio and television as well as local television and radio stations. This cultivated a mutually beneficial relationship in which we would be available to provide expert input on news pieces, meanwhile being able to more widely advertise our work in media coverage, such as our mosque vaccination work. For example, we worked with local families who had lost loved ones to COVID‐19 infection and journalists on news pieces to highlight the risk of COVID‐19 infection in ethnic minority communities.

## 7 Ramathan Campaign

We pre‐empted concerns around whether receiving vaccinations during the month of Ramathan may cause some concern among Muslims. Therefore, in addition to our work detailed above, we created specific videos delivered by doctors, scientists and faith leaders to encourage people to receive their vaccinations and reassure that this would not impact their ability to continue fasting. We also continued to host mosque vaccination pop‐ups during which several Imams received their vaccinations while fasting, who kindly agreed to film videos encouraging others to do the same. In addition to sharing educational videos and hosting webinars specific to vaccination during Ramathan, we also promoted general wellbeing and health advice for individuals observing Ramathan; this has become an annual event in the MDC calendar and continues to be well attended.

## 8 Summary

One member of our team was invited to join Team Halo, which was part of the United Nation's Vaccine Confidence Project, to join a team of healthcare professionals and scientists on social media to dispel vaccine myths. These videos and webinars were far reaching, including to audiences in the US and Australia.

MDC's grassroots approach, cultural sensitivity, and multilingual outreach were instrumental in addressing vaccine misinformation and hesitancy in Wales and beyond. Our work has been commended by politicians and health officials, highlighting the importance of community‐led initiatives in public health campaigns. This work highlighted the need for a grassroots approach to tackling dis‐ and misinformation and improving health education in a wider healthcare scope.

Our work has demonstrated that community‐based strategies delivered by trusted voices in trusted spaces are an effective approach to improving health outcomes and reducing inequalities in underrepresented communities. These approaches work because they are:

1. Culturally Relevant: Trusted community members understand the cultural, religious, and social contexts of their communities, allowing them to communicate health information in a respectful and relatable way.
2. Language Accessible: By using the preferred languages of the community, these strategies break down communication barriers and ensure that vital information is clearly understood. Using short videos and face‐to‐face interactions breaks down barriers created by lack of health literacy.
3. Trust Building: People are more likely to listen to and act on health advice when it comes from someone they know and trust—such as local faith leaders, doctors, or community figures—rather than distant institutions.
4. Location Sensitive: Delivering healthcare services and information in familiar, accessible places like mosques, churches, community centers, or local shops increases engagement and comfort levels. These settings also provide a safe space for individuals to share their concerns more openly.
5. Responsive and Inclusive: These strategies can be adapted quickly based on feedback and specific community needs, ensuring that no group is overlooked.

In short, misinformation during the COVID‐19 pandemic fueled fear, eroded trust, and led to behaviors that put both individuals and communities at greater risk. In order to improve preparedness for future pandemics, governments and health authorities must do more to tackle health inequalities faced by BAME and other vulnerable communities and better engage with these communities in order to build trust. Community‐based interventions help close the gap in health inequalities and empower underserved communities to facilitate informed decision making by addressing the root causes of mistrust and removing practical barriers. These are often, as in our case with MDC, delivered by volunteers. This is not a sustainable practice and more needs to be done to entrench these community‐based efforts into formalized health and education policy, in order to tackle health inequities at the root.

## Author Contributions

**Amara Naseem:** data curation. **Farzana Mohammed:** data curation. **Bnar Talabani:** conceptualization, investigation, writing – original draft, methodology, visualization, writing – review and editing, validation, formal analysis, project administration, supervision, resources. **Sayma Hanif:** conceptualization, methodology. **Emaad Alauddin:** software. **Kasim Ramzan:** conceptualization, investigation.

## Conflicts of Interest

The authors declare no conflicts of interest.

## Data Availability

Data sharing not applicable to this article as no datasets were generated or analysed during the current study.
